# The utility of ^68^Ga-DOTATATE and ^18^F-FDG PET/CT in predicting the response to tyrosine kinase inhibitors in patients with advanced medullary thyroid cancer

**DOI:** 10.1186/s13044-025-00250-x

**Published:** 2025-06-17

**Authors:** Eline C. Jager, James McNeil, Alexander J. Papachristos, Mark Sywak, Stan B. Sidhu, Rhonda Siddall, Jeremy Hoang, Geoffrey P. Schembri, Venessa H. M. Tsang, Ayanthi Wijewardene, Lyndal Tacon, Bruce Robinson, Roderick J. Clifton-Bligh, Martyn Bullock, Adrienne H. Brouwers, Thera P. Links, Schelto Kruijff, Anthony R. Glover, Matti L. Gild

**Affiliations:** 1 Department of Endocrinology and Diabetes, Royal North Shore Hospital, Sydney, Australia; 2 Department of Internal Medicine, Division of Endocrinology, University Medical Centre Groningen, Groningen, The Netherlands; 3 Department of Surgery, Division of Surgical Oncology, University Medical Centre Groningen, Groningen, The Netherlands; 4 Faculty of Medicine and Health, University of Sydney, Sydney, Australia; 5 Department of Endocrine Surgery, Royal North Shore Hospital, Sydney, Australia; 6 Department of Nuclear Medicine, Royal North Shore Hospital, Sydney, Australia; 7 Cancer Genetics Laboratory, Kolling Institute of Medical Research, Royal North Shore Hospital, Sydney, Australia; 8 Department of Nuclear Medicine and Molecular Imaging, University of Groningen, University Medical Centre Groningen, Groningen, The Netherlands; 9 Department of Molecular Medicine and Surgery, Karolinska Institutet, Stockholm, Sweden

**Keywords:** Medullary thyroid carcinoma, PET/CT imaging, Tyrosine kinase inhibitors, Response prediction

## Abstract

**Background:**

Tyrosine kinase inhibitors (TKIs) have significantly improved the prognosis of patients with advanced medullary thyroid cancer (MTC). However, treatment response heterogeneity leads to challenges in predicting individual favourable response. This study evaluated the correlation between PET metrics on ^68^Ga-DOTATATE and ^18^F-FDG PET/CTs prior to treatment, and TKI response.

**Methods:**

This study retrospectively evaluated patients with metastatic MTC who received TKIs at a tertiary care hospital and had prior ^68^Ga-DOTATATE and/or ^18^F-FDG PET/CT imaging. Patient demographics, treatment and PET/CT data were collected. Standardized Uptake Value (SUV) max, SUVmean, Total Lesion Activity (TLA) and Metabolic Tumor Volume (MTV) were determined per PET/CT.

**Results:**

In the 25 patients, mean age at diagnosis was 48 (±15) years; 11 (44%) were female and 21 tumors harbored *RET* driver alterations. Thirty-six TKI treatments were administered (11 patients received two TKIs sequentially). RECIST response rates (available in 32/36 treatments) included; stable disease in 8/32 (25%), partial response in 23/32 (72%) and complete response in 1/32 (3%) treatments. In total, 30 pre-TKI PET/CTs (24 ^68^Ga-DOTATATE PET/CTs, 6 ^18^F-FDG PET/CTs) were performed. Pre-TKI ^68^Ga-DOTATATE PET/CTs did not correlate with TKI treatment response. In the ^18^F-FDG cohort, high MTV and TLA correlated with a better structural response (*p* < 0.001) and high SUVmean correlated with a longer time to reach optimal response (*p* = 0.037).

**Conclusions:**

In a small cohort of MTC patients, MTV and TLA on ^18^F-FDG PET/CT were associated with the structural response of TKI treatment, suggesting their potential utility in identifying patients who are likely to respond significantly. In contrast, TKI response showed no correlation with uptake on ^68^Ga-DOTATATE PET/CT.

## Introduction

Medullary thyroid carcinoma (MTC) treatment has been revolutionised in the past decade with the advent of tyrosine kinase inhibitors (TKIs). The ascension of selective inhibitors for *RET*-mutant disease has further improved the treatment landscape [1]. Prior to the TKI era, the 10-year survival rate for patients with metastatic MTC was only 31%, with therapeutic options limited to palliative surgery and radiotherapy [2]. Substantial advancements in the understanding of the genetic mutations and signalling pathways in MTC, have resulted in the emergence of mutation-targeted therapy [3]. TKIs inhibit intracellular signal transduction pathways by blocking abnormally activated tyrosine kinase receptors [4, 5]. Multikinase inhibitors (MKI) lenvatinib, vandetanib and cabozantinib target multiple kinases, including vascular endothelial growth factor receptor (VEGFR) and epidermal growth factor receptor (EGFR), in addition to RET [5, 6]. While they have limited efficacy, adverse events are significant, due to off-target effects [7, 8]. RET-specific inhibition by selpercatinib has shown greater response rates and better tolerability which has led to selpercatinib being the TKI of choice for *RET*-mutant MTC [1, 9].

While TKIs have broadened the therapeutic landscape for MTC patients, determining the optimal timing for their initiation remains a challenge. Some patients, despite having significant disease burden, may be relatively asymptomatic, raising the question of whether systemic therapy should be started. This decision is further complicated by the potential for dose-limiting adverse events that could negatively impact quality of life and the potential development of resistance to TKIs. Additionally, the response to TKIs is heterogenous, with some patients experiencing a quick and durable response, while others do not [10].

Molecular imaging techniques may guide initial tumour staging and diagnose metastatic or recurrent disease. 2-deoxy-2-[fluorine-18]fluoro-D-glucose (^18^F-FDG) and ^68^Ga-DOTA-conjugated peptide (Tyr3)-octreotate (^68^Ga-DOTATATE) are commonly used PET tracers to detect MTC with mixed sensitivities [11]. ^18^F-FDG accumulates in cancer cells in proportion to their glycolytic activity and has a pooled sensitivity of 56% in MTC patients. However, sensitivity is improved in dedifferentiated tumors with short calcitonin-doubling times [12–14]. ^68^Ga-DOTATATE binds to somatostatin receptors (predominantly SSTR2), which are overexpressed on neuroendocrine tumour cells, modulating cell proliferation and secretory activity [15]. ^68^Ga-DOTATATE PET/CT has a pooled sensitivity of 64% [16] and is described as superior to ^18^F-FDG PET/CT, although available literature is limited [17, 18].

In neuroendocrine tumours (NETs), PET has been valuable in risk stratification and guiding treatments, showing potential for similar developments in MTC [19, 20]. PET imaging can be applied in any phase of the disease and provide real-time information. Improved prediction of TKI treatment response at the outset could improve individualized treatment, refine patient expectations and prevent side-effects in limited-response patients with this very rare disease. Moreover, suitable candidates for neo-adjuvant TKI treatment could be identified [21].

Here, we aimed to evaluate the relationship of pre-TKI ^68^Ga-DOTATATE and ^18^F-FDG PET/CT and treatment response in MTC patients undergoing TKI treatment. To our knowledge, this is the first study to report the correlation between PET metrics from both tracers and structural response to TKIs.

## Materials and methods

### Design and patients

**Fig. 1:**
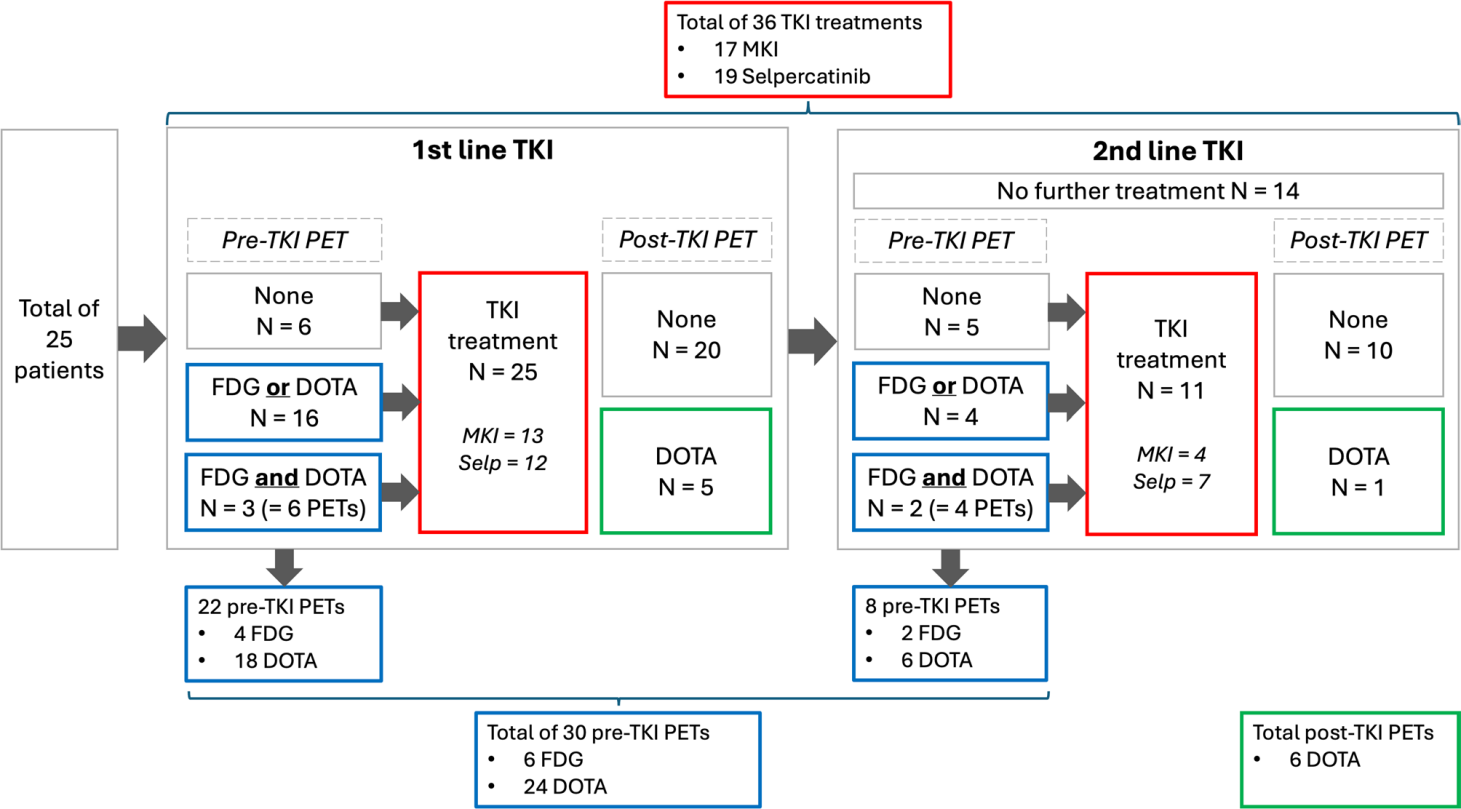
Flow diagram displaying the number of treatments and PET/CTs in the total of 25 included patients. Read from left to right: 25 patients were included, 22 PET/CTs were performed prior to first-line TKI treatment and five after TKI treatment had initiated. Eleven of 25 patients went on to receive second-line TKI treatment, which was preceded by eight PET/CTs. One patient had a PET/CT after second-line TKI treatment. Therefore a total of 30 PET/CTs were performed prior to TKI treatment. All patients without a PET/CT prior to first-line treatment, did have a PET/CT prior to second-line treatment, and vice versa. Therefore all 25 patients had at least one PET/CT prior to first- and/or second-line TKI treatment. Abbreviations: N = number of patients, TKI = tyrosine kinase inhibitor, PET = positron emission tomography, FDG = ^18^F-FDG PET/CT, DOTA = ^68^Ga-DOTATATE PET/CT, MKI = multikinase inhibitor, Selp = selpercatinib

MTC patients who received a TKI between 2013 and 2023 at a tertiary referral center were retrospectively evaluated and included when they had a ^68^Ga-DOTATATE and/or ^18^F-FDG PET/CT before the initiation of TKI therapy (Fig. 1). Patient and treatment characteristics were extracted from medical records and research databases. Ethics approval was granted by Northern Sydney Local Health District Human Research Ethics Committee (2024/ETH00393).

### Structural response to TKI

A diagnostic CT was performed at baseline and after TKI initiation, as part of standard clinical practice, to allow a response evaluation. The structural response to each TKI was reported according to Response Evaluation Criteria in Solid Tumors (RECIST) v1.1 for CT imaging, by dedicated radiologists [22]. The sum of target lesions at baseline (RECIST score baseline) and at the (first) nadir (RECIST score nadir), as well as the best RECIST classification (RECIST score classification) were recorded. In short, the RECIST score classifications include: disappearance of all target lesions (complete response [CR]), ≥ 30% decrease in the sum of the longest diameter of the target lesions (partial response [PR]), no PR or progressive disease (stable disease [SD]) and ≥ 20% increase in the sum of the largest diameters of the target lesions since start of treatment (progressive disease [PD]). The structural response was defined by the absolute change (RECIST score change = sum of target lesions at nadir – sum of target lesions at baseline), and the percentage change (RECIST score % change = [sum of target lesions at nadir – sum of target lesions at baseline] / sum of target lesions at baseline x 100) at the RECIST nadir, relative to the baseline RECIST score. The progression-free survival per treatment was defined by the time between start of treatment and RECIST progression (PD).

### Biochemical response to TKI

The calcitonin concentration at baseline (prior to TKI initiation) and at the nadir during TKI treatment were recorded per patient, per TKI treatment. The Siemens Immulite 2000 XPi immunoassay system was used to determine serum calcitonin concentrations. Calcitonin change was defined as the absolute difference between the baseline and nadir value, while calcitonin % change described the percentage decrease relative to the baseline value.

### Imaging acquisition

All images were acquired on a PET/CT with Time-of-Flight (ToF) capabilities and 21.6 cm axial field of view (Biograph mCT.S/64 PET/CT, Siemens Healthcare, Hoffman Estates, USA). Data were acquired from skull to mid-thigh. For ^68^Ga-DOTATATE PET/CT scans, subjects were injected with 120–180 MBq of ^68^Ga-DOTATATE produced in-house with imaging commencing approximately 50 min after injection. For ^18^F-FDG scans, subjects fasted for at least 6 h prior to tracer injection. Blood glucose levels were assessed to ensure they ranged 4–11 mmol/L. Subjects were administered 250 MBq of ^18^F-FDG if their weight was ≤ 90 kg or 300 MBq if > 90 kg, with imaging commencing approximately 60 min after injection. All PET reconstructions were performed using 3D OSEM with 2 iterations and 21 subsets.

### Image analysis

A dedicated thyroid cancer researcher (E.C.J.) and a nuclear physician (J.M) analysed the PET/CT scans using MIM software (MIM, version 7.1.3., MIM Software Inc.). Lesion selection and contouring were performed utilising a semi-automatic method. For ^68^Ga-DOTATATE PET/CTs and ^18^F-FDG PET/CTs, a flat SUV threshold of 2.5 and 3.0 were used, respectively, for the initial selection of lesions. All lesions were manually reviewed to exclude physiological or inflammatory uptake. For indeterminate lesions, the expert opinion of another nuclear physician (J.H.) was obtained to reach consensus. Avid liver lesions above the threshold were manually contoured.

The following PET parameters were determined per patient and per region (nodal, bone, lung, liver); Standardized Uptake Values (SUVmax and SUVmean), Total Lesion Activity (TLA) and Metabolic Tumor Volume (MTV). MTV was calculated as the sum of metabolic volumes of all tumor lesions. Strictly speaking, ^68^Ga-DOTATATE is a receptor-density tracer, and not a metabolic tracer, however, for simplicity, MTV was used for both types. TLA was calculated as the metabolic activity of total tumor lesions (SUVmean*MTV). MIM software performed automatic calculation of these PET metrics.

### Statistical analysis

Frequencies with percentages were used to report categorical data, and medians with interquartile or mean with standard deviation (depending on distribution) described continuous data. Fisher’s exact test identified differences between groups in categorical data. The independent-samples t-test or Mann-Whitney U test was used to assess differences between groups in continuous data (depending on normality of distribution). Correlations between parameters were established with Spearman’s rho correlation coefficient. We constructed Kaplan Meier curves to evaluate survival differences between groups. All analyses were performed in IBM Statistics Version 29. Graphs were produced in Prism GraphPad Version 9. A p-value of < 0.05 was considered statistically significant.

## Results

### Patient characteristics

A total of 25 patients with an ^18^F-FDG PET/CT and/or ^68^Ga-DOTATATE PET/CT prior to TKI treatment were identified (Fig. 1; Table 1). Mean age at diagnosis was 48 (±15) years, and 11 patients (44%) were female. Three patients (12%) had germline mutations consistent with Multiple Endocrine Neoplasia Type 2 A (MEN2A) and 18 patients (72%) harboured somatic *RET* alterations. In 4 patients, presence of a somatic *RET* alterations was not assessed.

**Table 1:** Patient and treatment characteristics

	All patients *n* = 25 *N* (%), mean (SD) or median (IQR)
Female sex	11 (44)
Age at diagnosis (y)	48 (±15)
MEN2	3 (12)
*RET*-mutated^1^	21 (84)
M918T	14 (67)
C634	3 (14)
C630R	1 (5)
C618A	1 (5)
C620G	1 (5)
p.632_633del	1 (5)
T – stage at diagnosis	
TX	2 (8)
T1-2	11 (44)
T3-4	12 (48)
N – stage at diagnosis	
N0	2 (8)
N1	23 (92)
M – stage at diagnosis	
MX	1 (4)
M0	14 (56)
M1	10 (40)
Previous total thyroidectomy	25 (100)
Size of primary tumor (mm)^2^	30 (21–50)
Previous lymph node dissection(s)	
1	14 (56)
≥ 2	11 (44)
Post-operative biochemical cure^2^	1 (4)
Distant metastasis at final follow-up^3^	24 (96)
Distant metastatic sites at final follow-up	
Bone	17 (68)
Lungs	14 (56)
Liver	14 (56)
Brain	3 (12)
Number of TKIs	
1	14 (56)
2	11 (44)
Type of TKI	
Vandetanib	4 (16)
Cabozantinib	8 (32)
Lenvatinib	5 (20)
Selpercatinib	19 (76)
Time to first TKI after diagnosis (mo)	47 (6–90)
Total follow-up time (mo)	98 (41–174)
Deceased at final follow-up	10 (40)

^1^In 4 (16%) patients no germline *RET* alteration was detected, however, the presence of a somatic *RET* alteration is unknown. ^2^Unknown in 2 patients. ^3^The only patient without distant metastasis had an extensive mediastinal mass requiring TKI treatment. Percentages may not sum to 100% due to rounding off. Abbreviations: MTC = medullary thyroid cancer, y = years, MEN2 = Multiple Endocrine Neoplasia Type 2A, *RET* = Rearranged during Transfection, mo = months, TKI = tyrosine kinase inhibitor

**Fig. 2:**
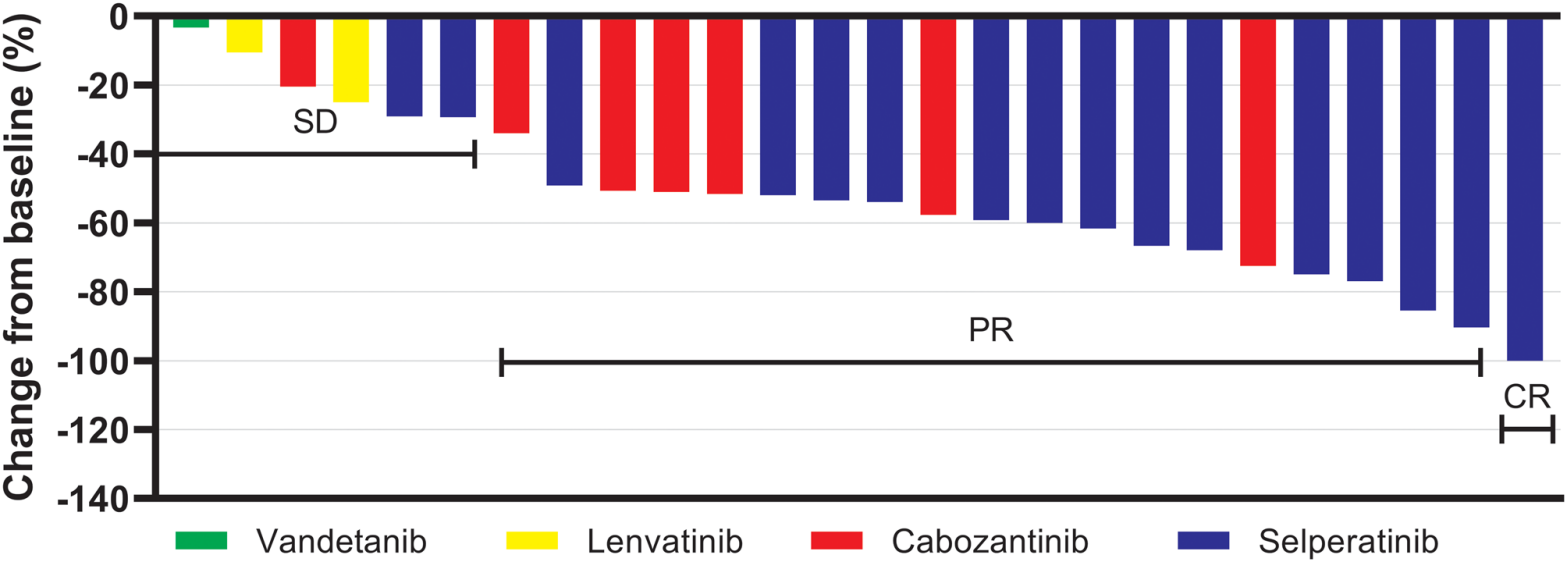
Waterfall plot displaying the percentage change in RECIST score during TKI treatment. Following each treatment event, the structural response was assessed. Of 36 treatments, 26 had available measurements of baseline and nadir target lesions and were included in the figure. Abbreviations: SD = stable disease, PR = partial response, CR = complete response

At diagnosis, 10 patients (40%) had distant metastasis, whereas 24 (96%) had distant metastasis at final follow-up (Table 1). The most common distant metastatic site was bone (68%). Fourteen patients (56%) received one TKI and 11 patients (44%) received two TKIs (always sequentially) (Figs. 1 and 2). The most commonly prescribed TKI was selpercatinib (19/25 patients, 76%). Lenvatinib was prescribed off-label. The median time between diagnosis and first TKI was 47 months (IQR 6–90). Of 25 patients, 24 had a ^68^Ga-DOTATATE PET/CT and 6 had a ^18^F-FDG PET/CT prior to TKI treatment (5 patients had both scans, see Fig. 1). At final follow-up, 10 patients (40%) had died due to MTC progression.

### TKI treatments

To document all TKI treatments in the 25 patients, each treatment was categorized as a distinct ‘treatment event’ (Table 2). A total of 36 treatment events were identified in the 25 patients (25 first-line and 11 s-line TKI treatments, see Fig. 1). Overall, the 36 treatments included 17 MKI treatments and 19 selpercatinib treatments. Seven of 19 selpercatinib treatments were preceded by MKI treatment. The best RECIST response (available in 32 treatments) was 8/32 (25%) stable disease, 23/32 (72%) partial response and 1/32 (3%) complete response (Fig. 2). Treatment duration varied widely (28 months, IQR 10–46). The best structural response on selpercatinib and MKI treatment was 63% (±20) and 38% (±22) decrease in the RECIST score, respectively (*p* = 0.003). The time to reach RECIST score nadir was 23 months (IQR 8–32) on selpercatinib treatment, and four months (IQR 2–8) on MKI treatment (*p* = 0.001). Selpercatinib treatment led to a 99.4% (IQR 98.8–99.8) reduction in calcitonin, in contrast to 90.3% (IQR 55.9–95.9) reduction on MKI treatment (< 0.001). The progression-free survival (PFS) was higher on selpercatinib (63%, 37 months IQR 10–51) than on MKI (30%, 10 months IQR 7–30) treatment (*p* = 0.011).

**Table 2:** Tyrosine kinase inhibitor (TKI) treatments events and response

	AD^*^	All treatments events *n* = 36	MKI *n* = 17	Selpercatinib *n* = 19	*P*-value
		*N*/AD^*^ (%), median (IQR) or mean (±SD)			
Treatment duration (mo)	35	28 (10–46)	15 (4–36)	43 (14–55)	0.018
*Structural response*					
RECIST classification	32				1.000
Stable disease (SD)		8/32 (25)	4/14 (29)	4/18 (22)	
Partial response (PR)		23/32 (72)	10/14 (71)	13/18 (72)	
Complete response (CR)		1/32 (3)	0/14 (0)	1/18 (6)	
RECIST score baseline (mm)	26	51 (±27)	51 (±35)	51 (±22)	0.988
RECIST score nadir (mm)	26	23 (±17)	31 (±20)	18 (±13)	0.054
RECIST score change (mm)	26	28 (±21)	20 (±21)	33 (±20)	0.134
RECIST score % change	26	53 (±24)	38 (±22)	63 (±20)	0.003
Time to RECIST nadir (mo)	26	8 (4–26)	4 (2–8)	23 (8–32)	0.001
*Biochemical response*					
Calcitonin baseline (pg/ml)	28	1380 (751–6605)	894 (236–3266)	1840 (1020–9850)	0.129
Calcitonin nadir (pg/ml)	28	24 (5–79)	81 (36–210)	8 (5–42)	0.002
Calcitonin change (pg/ml)	28	1352 (718–6596)	773 (183–3076)	1835 (1015–9832)	0.105
Calcitonin % change	28	99.0 (93.7–99.6)	90.3 (55.9–95.9)	99.4 (98.8–99.8)	< 0.001
Time to calcitonin nadir (mo)	28	4 (1–21)	1 (1–5)	7 (1–30)	0.142
*Disease progression*					
Progression-free survival	26	13/26 (50)	3/10 (30)	10/16 (63)	0.011
Time to RECIST progression (mo)	25	24 (9–47)	10 (7–30)	37 (10–51)	0.065

Not all response parameters were available for each treatment event ^*^AD = available data per variable; frequencies with percentages are reported relative to the available data of that each variable. Percentages may not sum to 100% due to rounding off. Abbreviations: MKI = multi-kinase inhibitor, IQR = interquartile range, SD = standard deviation, mo = months, TKI = tyrosine kinase inhibitor

### Response prediction based on pre-TKI ^68^Ga-DOTATATE PET/CT

**Fig. 3:**
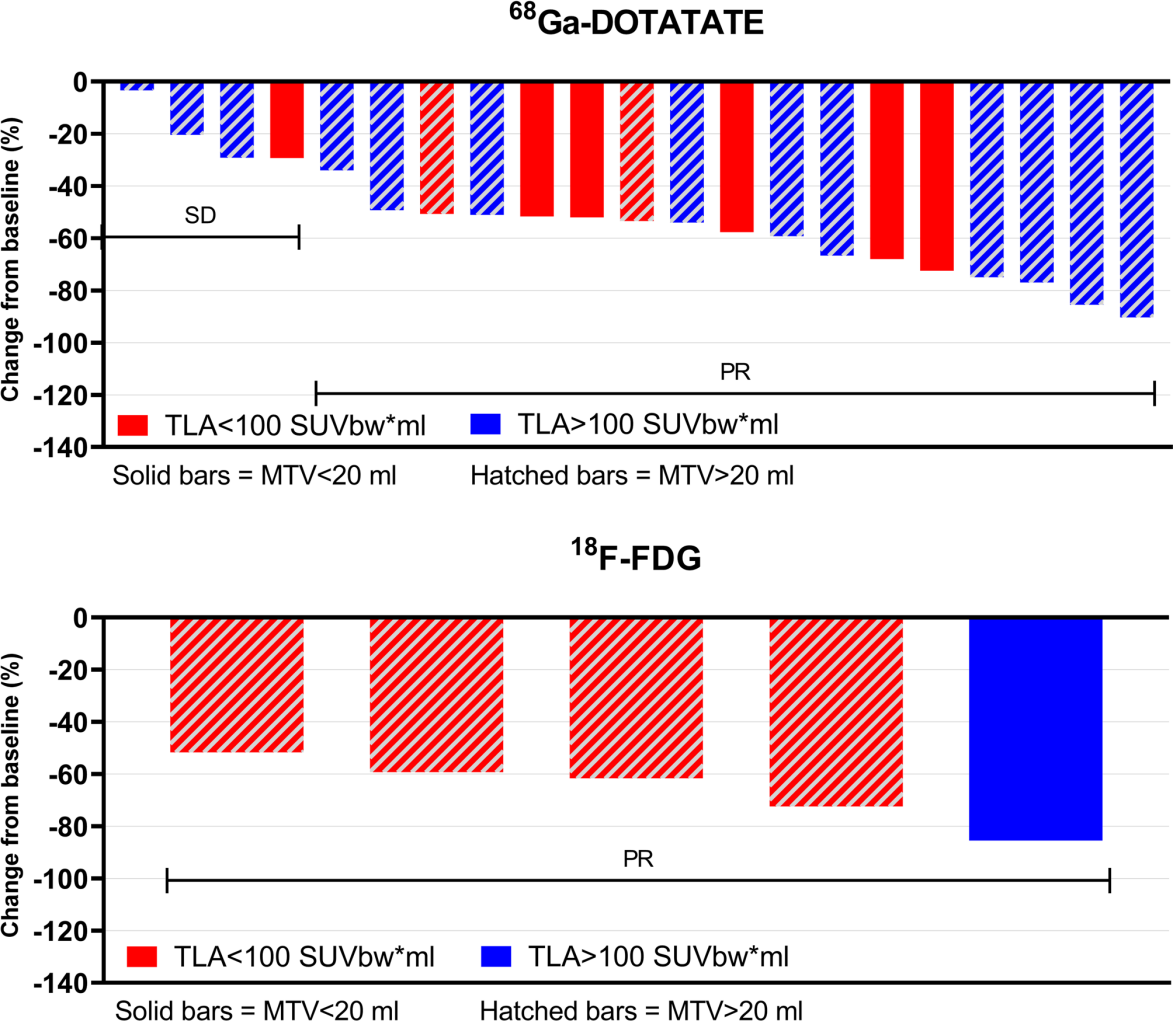
Waterfall plot displaying the percentage change in RECIST score during TKI treatment stratified by TLA (SUVbw*ml) and MTV (ml) score on pre-TKI ^68^Ga-DOTATATE PET/CT (top graph) and pre-TKI ^18^F-FDG PET/CT (bottom graph). RECIST score % change was available in 21 of 24 patients with a pre-TKI ^68^Ga-DOTATATE PET/CT and in 5 out of 6 patients with a pre-TKI ^18^F-FDG PET/CT. Abbreviations: SD = stable disease, PR = partial response

**Fig. 4:**
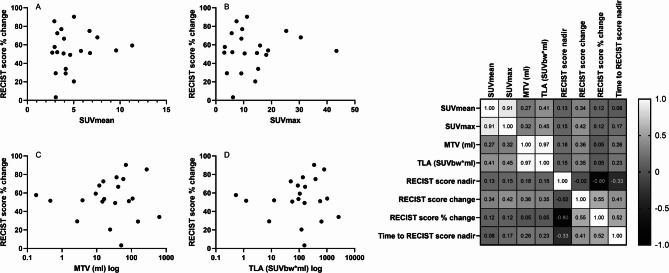
Left: Scatterplots displaying the correlations between PET parameters on pre-TKI ^68^Ga-DOTATATE PET/CT and RECIST score % change (**A** – **D**) during tyrosine kinase inhibitor treatment. The RECIST score % was available in 21 of 24 patients with ^68^Ga-DOTATATE PET/CT. Right: Correlation matrix showing the correlations between structural response measures and PET parameters for ^68^GA-DOTATATE PET/CT. No significant correlations were identified

We evaluated whether PET metrics on pre-TKI ^68^Ga-DOTATATE PET/CT correlated with treatment response. Median time between ^68^Ga-DOTATATE PET/CT and TKI treatment was four months (IQR 2–8). On all 24 pre-TKI ^68^Ga-DOTATATE PET/CTs, a median of eight (IQR 5–16) ^68^Ga-DOTATATE avid lesions were detected. Avid nodal, bone, lung and liver lesions were seen on 28, 16, 2 and 7 ^68^Ga-DOTATATE PET/CTs. Median SUVmean, SUVmax, MTV and TLA was 4.1 (IQR 3.2–5.7), 10.8 (IQR 6.5–15.7), 34.1 ml (11.9–69.2) and 145.7 SUVbw*ml (IQR 65.1–491.7), respectively. In 21/24 patients with ^68^Ga-DOTATATE PET/CTs, the structural response is shown (Fig. 3). No significant correlations between SUVmean, SUVmax, MTV or TLA and structural or biochemical response parameters were identified (Fig. 4). However, high SUVmean and SUVmax in bone lesions, specifically, did correlate with a better overall structural response (i.e. higher RECIST score change of non-bone lesions) (r^2^ = 0.609 *p* = 0.027, and r^2^ = 0.576 *p* = 0.039, respectively). No predictive value for PFS or OS was identified in the whole cohort, nor in the subset of selpercatinib-treated patients.

### Response prediction based on pre-TKI ^18^F-FDG PET/CT

**Fig. 5:**
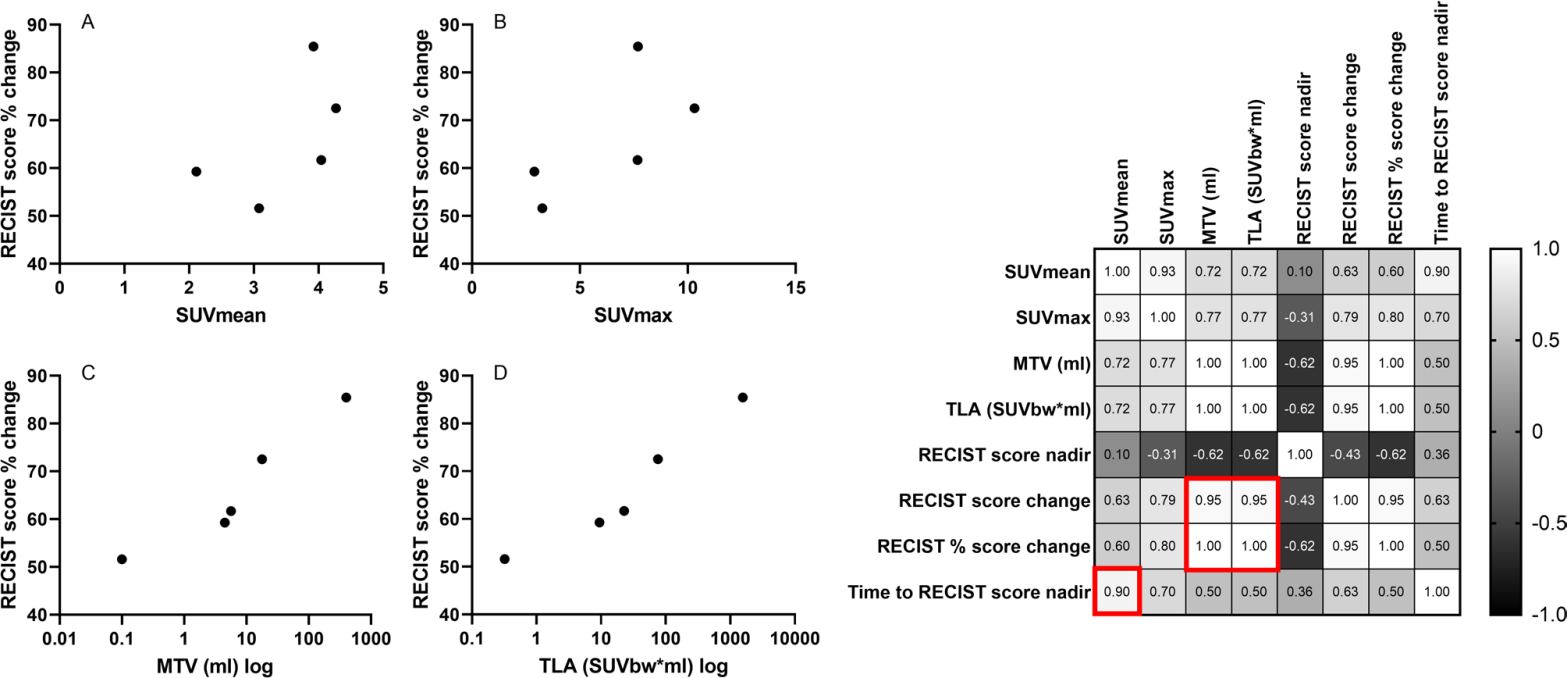
Left: Scatterplots displaying the correlations between PET parameters on pre-TKI ^18^F-FDG PET/CT and RECIST score % change (**A** – **D**) during tyrosine kinase inhibitor treatment. The RECIST score % was available in five of six patients with ^18^F-FDG PET/CT. Right: Correlation matrix showing the correlations between structural response measures and PET parameters for ^18^F-FDG PET/CT. The red boxes indicate significant correlations

We also analyzed pre-TKI ^18^F-FDG PET/CTs (available in six patients), which were performed six months (IQR 4–8) prior to TKI treatment. A median of eight (IQR 1–29) ^18^F-FDG avid lesions were detected. The median SUVmean, SUVmax, MTV and TLA was 4.0 (IQR 2.8–4.3), 7.7 (3.2–10.0), 11.7 ml (IQR 3.4–406.5) and 49.2 SUVbw*ml (7.1–1630.4), respectively. Avid lesions were identified in lymph nodes (5/6 patients), bone (2/6 patients), lungs (2/6 patients) and liver (2/6 patients). Of the five patients in whom data on a structural response was available (Fig. 3), high MTV and TLA correlated with a higher RECIST score change (r^2^ = 0.95 *p* = 0.014, r^2^ = 0.95 *p* = 0.014, respectively) and with RECIST score % change (r^2^ = 1.00 *p* < 0.001, r^2^ = 1.00 *p* < 0.001) (Fig. 5). In addition, a high SUVmean correlated with a longer time to reach RECIST nadir (r^2^ = 0.90, *p* = 0.037). In 3/6 patients with selpercatinib treatment and available structural response data, the positive correlation between MTV or TLA and structural response remained, while a positive correlation with SUVmean and SUVmax also appeared (both r^2^ = 1.000, *p* < 0.001). No significant correlations between PET metrics on ^18^F-FDG PET/CT and biochemical response measures were identified. Low numbers impeded sub-analyses of avid lesions by location and reliable survival analyses.

### Pre- and post-TKI ^68^Ga-DOTATATE PET/CT

**Fig. 6:**
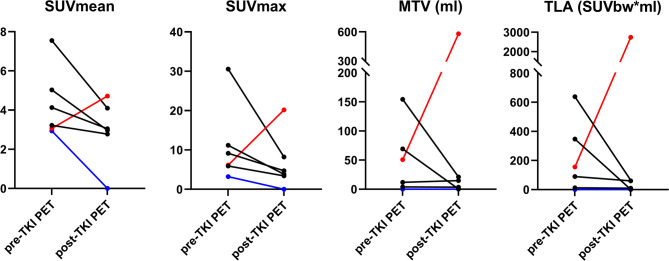
Changes in PET parameters between pre-TKI and post-TKI ^68^Ga-DOTATATE PET/CTs. Red: vandetanib, blue: cabozantinib, black: selpercatinib. Abbreviations: SUV = standardized uptake value, MTV = metabolic tumor volume, TLA = total lesion activity, TKI = tyrosine kinase inhibitor

**Fig. 7:**
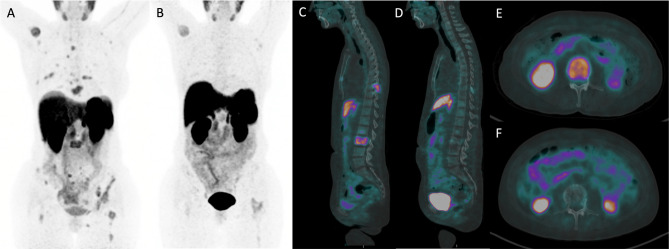
^68^Ga-DOTATATE PET/CT images displaying tracer uptake changes in a patient treated with selpercatinib. **A**, **C**, **E** = pre-TKI ^68^Ga-DOTATATE PET/CT. **B**, **D**, **F** = post-TKI ^68^Ga-DOTATATE PET/CT

Pre- and post-TKI ^68^Ga-DOTATATE PET/CT scans were available for six patients. Of these, four patients were on selpercatinib, one patient was on cabozantinib and one patient was on vandetanib. In the preliminary evaluation of ^68^Ga-DOTATATE PET/CT as a monitoring tool during treatment, changes in ^68^Ga-DOTATATE PET metrics were determined. The median percentage decrease in SUVmean, SUVmax, MTV and TLA from pre- to post-TKI ^68^Ga-DOTATATE PET/CT were 33% (IQR 17–45), 57% (IQR 44–71), 48% (-15–96%) and 61% (IQR 25–97) (Fig. 6). In the four selpercatinib-treated patients, all PET metrics decreased from pre- to post-TKI ^68^Ga-DOTATATE PET/CT, only MTV increased slightly in one patient. A selpercatinib-treated patient with the most notable response for all PET parameters is presented in Fig. 7.

## Discussion

As TKIs become more available and effective, understanding the heterogeneous response in metastatic MTC is crucial for guiding treatment decisions and advancing personalized medicine. In this retrospective study, the predictive value of avidity metrics on ^18^F-FDG and ^68^Ga-DOTATATE PET/CT was evaluated in a unique TKI-treated cohort of MTC patients. High ^18^F-FDG MTV and TLA were shown to herald a better response to TKI treatment than low MTV and TLA in a small cohort of patients. Evaluation of ^68^Ga-DOTATATE PET/CTs highlighted considerable heterogeneity in avidity markers while no correlation with TKI treatment outcomes was identified.

This study identified a strong positive correlation between MTV and TLA on ^18^F-FDG PET/CT and subsequent RECIST response to TKI treatment in a small cohort of patients with this rare disease. In the limited subgroup of selpercatinib-treated patients, this correlation remained significant, suggesting better RET inhibition in the presence of a high tumor burden with high glycolytic activity. This study is the first to extrapolate the correlation between MTV and TLA on ^18^F-FDG PET/CT and a response to TKI. Our findings may be contradictory to previously published data by Werner et al. on the predictive value of SUVmean and SUVmax on ^18^F-FDG PET/CT in MTC patients treated with vandetanib. Their findings suggest that high metabolic disease activity (SUVmean > 4) on ^18^F-FDG PET/CT predicts a shorter PFS. In addition, a sustained high SUVmean during vandetanib treatment was related to a worse prognosis [23, 24]. Another study in nineteen patients with well-differentiated thyroid cancer and three patients with MTC did not identify a correlation between baseline SUV and response to sunitinib [25]. While our study is the first to include mostly selpercatinib-treated patients, which may have different outcomes due to their selective action, our findings indicate a better structural response in the presence of high baseline tumor burden and metabolic disease activity (MTV and TLA). The results seem contradictory since a better structural response is generally associated with a better prognosis, particularly for RET-inhibitors. On the other hand, the positive correlation between SUVmean and time to reach optimal TKI response suggests that a higher baseline metabolic activity, independent of tumor burden, may delay the therapeutic effect of TKIs, necessitating longer treatment duration to achieve optimal outcome. Our findings in this small cohort, with somewhat contradictory results, might therefore be coincidental observations and limit definitive conclusions.

In contrast, ^68^Ga-DOTATATE avidity was heterogeneous and uncorrelated with a response to TKI treatment. As such, therapeutic responses in MTC patients with large ^68^Ga-DOTATATE avid disease burden and highly ^68^Ga-DOTATATE avid disease may be comparable to those with low tumor burden or avidity. Previous studies have shown that ^68^Ga-DOTATATE avidity is lower in MTC than in other NETs. Moreover, ^68^Ga-DOTATATE avidity on PET/CT imaging is not directly related to immunohistochemical expression of SSTR type 2 or 5 [26, 27], which can be a result of tumor heterogeneity and functional receptor availability affecting in vivo versus static in vitro measurements. Nevertheless, ^68^Ga-DOTATATE PET/CT can be utilized for mapping the extent of metastasis in MTC [28, 29], especially in the presence of high circulating calcitonin and bone metastases [30–33].

In evaluating the role of ^18^F-FDG and ^68^Ga-DOTATATE PET/CT in predicting TKI response, it is crucial to consider the insights these imaging modalities provide into the underlying tumor biology. High ^18^F-FDG uptake reflects increased glucose metabolism, typically a hallmark of aggressive, poorly differentiated tumors with high proliferative activity and reliance on glycolysis, often driven by hypoxia-inducible factors and angiogenic pathways [13, 34, 35]. Such tumors may therefore be more sensitive to TKIs, which inhibit VEGFR and related signaling cascades critical for survival and progression [6]. Conversely, high uptake on SSTR-targeted PET/CT is more typical for well-differentiated tumors [36], possibly signifying lower metabolic rates and proliferative activity, and less dependence on angiongenesis and hypoxia-driven mechanisms. These SSTR-expressing tumors are potentially less influenced by mechanisms targeted by TKI therapy, which could explain the lacking correlation between ^68^Ga-DOTATATE PET/CT and TKI response in our study.

Selpercatinib is the first treatment choice in advanced, *RET*-altered MTCs, with a 12-month PFS of 87%, compared to 66% for MKIs [37]. Retrospective review of selpercatinib-treated patients in this study, when compared to MKIs, showed better response rates, though lack of prospective randomization limits unbiased interpretation. Interestingly, the optimal structural response occurred later in the selpercatinib cohort than in the MKI cohort, which may hypothetically be an effect of pre-treatment with an MKI (7/19 selpercatinib patients) [38].

MTC has long been recognized as an elusive tumor with substantial heterogeneity in its clinical course. Many efforts to predict patient outcomes of this rare disease have led to the 2021 publication of the International Medullary Thyroid Cancer Grading Scheme (IMTCGS) [39]. The IMTCGS allows accurate prognostication post-surgery but lacks the capacity to predict prognosis when influenced by treatments and is therefore a static prognosticator. As TKI use increases, additional tools are required to predict treatment response and ascertain the optimal timing for commencement.

The choice for a RET-specific inhibitor TKI is based on the presence of a *RET* alteration, determined in a tumor biopsy. However, biopsies are usually obtained from a single lesion, while patients may have multiple lesions with considerable molecular and tumor microenvironment heterogeneity. PET imaging may provide non-invasive information of individual lesions, throughout various phases of the disease. In NETs, Binderup et al. has already shown the superiority of ^18^F-FDG PET/CT to static WHO grading in risk stratification and treatment response to PRRT, showing potential for similar developments in MTC [20]. In addition, the NETPET score and total discordant avidity volume between ^18^F-FDG PET/CT and ^68^Ga-DOTATATE PET/CT in NETs highlight the prognostic and complementary roles of multiple PET tracers [19, 40], encouraging similar evaluations in large cohorts of MTC patients.

Without evidence-based guidelines for PET imaging in MTC patients, the choice for a specific PET tracer is based on costs, availability and clinician preference. While ^18^F-DOPA PET/CT is ideal for mapping MTC [11, 28], it is not available in all centers due to a complex production process and high costs. ^68^Ga-DOTATATE PET/CT is an alternative for localizing neuroendocrine tumors including MTC. In contrast, ^18^F-FDG is widely available and its production is well-established, relatively simple, and more cost-effective due to high demand for various indications. ^18^F-FDG PET/CT is often performed in selected MTC patients with suspected dedifferentiated tumors with short calcitonin-doubling times or when ^18^F-DOPA and ^68^Ga-DOTATATE are unavailable [13, 41], explaining the paucity of ^18^F-FDG PET/CTs available for this study.

Our study is the first to assess the correlation between PET metrics on both ^68^Ga-DOTATATE and ^18^F-FDG PET/CT and the RECIST response to various TKIs in advanced MTC patients. Prior studies have primarily focused on extrapolating the sensitivity of either tracer for MTC lesions. Limitations of this study are the retrospective nature, including absent data and the small number of patients, particularly for the ^18^F-FDG PET/CT cohort. As such, these findings limit biased conclusions and should be taken as single observations. The small cohort is largely a result of the rarity of this disease and inclusion criteria. In addition, the time interval between pre-TKI PET/CTs and start of treatment was quite variable, since a pre-TKI PET/CT is not standard practice. Moreover, adverse events were not consistently reported impeding correlative assessment with PET metrics. Preliminary analysis of PET metrics on the available pre- and post-TKI ^68^Ga-DOTATATE PET/CT suggests a decrease in avidity measures during treatment. However, SUVs are not completely reliable for monitoring receptor density during treatment because they reflect total uptake, which may be influenced by factors like blood flow and receptor saturation. Future studies with larger prospective cohorts and standardized intervals between PET/CT imaging and TKI treatment are needed to validate these findings. In addition, selection bias should be limited by performing PET/CT with a tracer of interest in all patients, prospectively. Finally, for adequate assessment of ^68^Ga-DOTATATE PET/CT as a monitoring tool during treatment, the tumor-to-blood ratio is considered a more accurate metric and could be used in a prospective study [42].

Determining the optimal timing for TKI treatments for individual patients with metastatic MTC remains challenging with limited evidence-based information available. Resistance to TKIs may be driven by several factors and it is not known if the timing of therapy initiation contributes to reduced long-term efficacy. Thus, using reliable predictive measures such as PET metrics and correlating with structural response to TKIs may highlight how clinicians could identify patients most likely to benefit and incorporate this in personalised decision making. In a small cohort of patients, we observed a potential role of ^18^F-FDG PET/CT in identifying patients likely to achieve a significant response to TKI treatment. However, the limited sample size and somewhat contradictory results, warrant further investigation in larger studies. Conversely, ^68^Ga-DOTATATE PET/CT appears to have utility primarily in disease mapping, with limited effectiveness in predicting TKI treatment response.

## Data Availability

The datasets used and/or analysed during the current study are available from the corresponding author on reasonable request.

## Abbreviations

MTC: Medullary thyroid cancer
RET: Rearranged during Transfection
TKI: Tyrosine kinase inhibitor
MKI: Multi-kinase inhibitor
VEGFR: Vascular endothelial growth factor receptor
EGFR: Epidermal growth factor receptor
^18^F-FDG: 2-deoxy-2-[fluorine-18]fluoro-D-glucose
^68^Ga-DOTATATE: ^68^Ga-DOTA-conjugated peptide (Tyr3)-octreotate
SSTR2: Somatostatin receptor 2
NET: Neuroendocrine tumor
RECIST: Response Evaluation Criteria in Solid Tumors
CR: Complete response
PR: Partial response
SD: Stable disease
PD: Progressive disease
ToF: Time-of-flight
SUV: Standardized uptake value
TLA: Total lesion activity
MTV: Metabolic tumor volume
MEN2A: Multiple endocrine neoplasia type 2 A
IQR: Interquartile range
